# Israeli medical students’ perceptions of six key medical specialties

**DOI:** 10.1186/2045-4015-2-19

**Published:** 2013-05-21

**Authors:** Charles Weissman, Howard Tandeter, Rachel Yaffa Zisk-Rony, Yoram G Weiss, Uriel Elchalal, Alex Avidan, Josh E Schroeder

**Affiliations:** 1 Department of Anesthesiology and Critical Care Medicine, Hadassah-Hebrew University Medical Center, Hebrew University Hadassah School of Medicine, POB 12000, Jerusalem 91120, Israel; 2 Department of Obstetrics and Gynecology, Hadassah-Hebrew University Medical Center, Hebrew University Hadassah School of Medicine, Jerusalem, Israel; 3 Department of Orthopedic Surgery, Hadassah-Hebrew University Medical Center, Hebrew University Hadassah School of Medicine, Jerusalem, Israel; 4 Henrietta Szold Hebrew University Hadassah School of Nursing, Jerusalem, Israel; 5 Department of Family Medicine, School of Continuing Medical Education Ben Gurion University School of Medicine, Be’er Sheva, Israel

**Keywords:** Medical education, Residency, Medical specialties, Medical students, Marketing research

## Abstract

**Background:**

Choosing a medical specialty requires medical students to match their interests and social-cultural situations with their perceptions of the various specialties.

**Objectives:**

Examine Israeli 6th-year medical students’ perceptions of six key specialties: pediatrics, orthopedic surgery, anesthesiology, obstetrics/gynecology, general surgery and family medicine.

**Methods:**

Questionnaires distributed to 355 6th-year students from three successive classes (2008–2010) of 6th-year students at the Hebrew University – Hadassah School of Medicine, Jerusalem, Israel and the 2010 class of the Ben Gurion University School of Medicine, Be’er Sheva, Israel.

**Results:**

Responses were obtained from 234 students, for a response rate of 66%. Pediatrics and obstetrics/gynecology were the specialties most often under positive career consideration by individual students. Anesthesiology and general surgery were least often under positive career consideration and were viewed as being in a workforce crisis. Pediatrics and family medicine, found to be especially popular among women, were perceived by 58% and 78% of respondents, respectively, as providing reasonable ratios of lifestyle to income. None of the students thought the same about general surgery and only 28% thought so about anesthesiology. Pediatrics and obstetrics/gynecology were reported to afford a controllable lifestyle by 63% and 8%, respectively, With respect to positive career considerations and lifestyle perceptions, there were no differences between the opinions of men and women students. Differences between genders arose in responses to queries of whether a specialty was interesting and challenging. Women were more likely than men to perceive pediatrics and family medicine as interesting and challenging while men were more likely to think that general and orthopedic surgery are interesting and challenging.

**Conclusions:**

Knowing the medical students’ perceptions of the various specialties should help in understanding the maldistribution of physicians among the various specialties. Such data can also be an important input into the efforts of the healthcare leadership to promote a specialty distribution that matches the population’s evolving needs.

## Background

Selecting a career is one of the most important decisions made during a lifetime. The decision requires that individuals match their interests, social situations and cultural backgrounds with a specific vocation. The individual generally gathers information about a number of careers from a variety of objective and subjective sources to form an opinion of the occupation [1]. Medical students must make a two or three-step career decision. The initial decision is the educational choice to become a physician and attend medical school [1]. The second decision is the occupational choice of one of the more than 20 medical specialties for residency training. A further step is deciding whether to pursue further training and, if yes, in which subspecialty.

The medical specialties differ greatly in work settings, skill sets, duties, responsibilities and professional interests, so that they essentially constitute a group of distinct occupations [2,3]. Therefore, choosing a specialty is complicated and made even more problematic by modern medical care becoming increasingly complex, resulting in increasing specialization and sub-specialization. Simultaneously, as populations continue to grow, so does the demand for more primary care physicians. The distribution of physicians among the primary, secondary and tertiary specialties is a function of the number of medical school graduates, their choice of specialties, and the number of available residency and fellowship training positions.

Israel is beset by a looming shortage of physicians, an inadequate number of medical school graduates and a maldistribution of physicians among the various specialties, with shortages in areas such as general surgery and anesthesiology. This problematic situation requires healthcare system leadership to gain additional insights into issues concerning the physician workforce, such as medical students’ perceptions of the various specialties. Knowing these perceptions is especially important for discerning why some specialties are more popular than others among the medical students.

This study is a component of a larger investigation of the processes Israeli medical students utilize when selecting medical specialties [4,5]. It specifically explores a key component in the second decision step enumerated above (i.e. decisions about which specialty to pursue), namely final (6th) year medical students’ perceptions of six key specialties: (pediatrics, orthopedic surgery, anesthesiology, obstetrics/gynecology, general surgery, and family medicine). The study examines student perceptions of both the professional and non-professional aspects of these specialties. The interest in the latter is due to the many studies showing the importance that the current generation of medical students attaches to balancing professional obligations with free-time (child-rearing, family time) and a controllable lifestyle with financial compensation [6-8]. The objective of the study was to better understand how and why medical students, both male and female, would choose or reject one of these specialties for residency training and career.

## Methods

The methods used in this study were adapted from marketing research methods and have been described elsewhere [5]. Briefly, this methodology proposes that when a consumer’s (i.e. student’s) criteria match his/her perceptions of a product’s (i.e. specialty) features, the likelihood of a purchase (selecting the specialty) increases. Therefore, to provide insights into the selection process, this study examined the two sides of the marketing equation: both the students’ selection criteria and their perceptions of the various specialties. This was accomplished using a questionnaire designed to elicit (1) the importance of each of the 25 criteria with respect to the students’ choice of a medical specialty; (2) their perceptions (16 items) of how six key specialties (pediatrics, orthopedic surgery, anesthesiology, obstetrics/gynecology, general surgery and family medicine) rate on each of the 25 criteria; (3) their level of consideration in pursuing a career in each of these specialties; and (4) demographic data. A 5-point Likert scale was used for answering the questions related to the first three items in this list.

The study focused on four specialties mirroring those practiced by the investigators (family medicine, obstetrics/gynecology, orthopedic surgery and anesthesiology) plus pediatrics and general surgery. This provided information on two primary care specialties (pediatrics and family practice) and two surgical specialties (general and orthopedic surgery) plus obstetrics/gynecology and anesthesiology. General Internal Medicine was not chosen because Internal Medicine is usually a “pass through” residency i.e. a way-station on the way to sub-specialization. Only a minority of Israeli internal medicine residents do not sub-specialize^a^.

The questionnaires underwent two pilot studies to examine ease-of-use and determine whether modifications were needed. Each of these initial studies included fifteen 6th-year medical students. During the pilot studies, information was elicited on 17 items for each of 10 specialties. However, many of the respondents failed to answer all the questions because of the excessive time it was taking to complete the forms. Therefore, the number of specialties was reduced to six to provide information on two primary care specialties (pediatrics and family practice) and two surgical specialties (general and orthopedic surgery) plus obstetrics/gynecology and anesthesiology [5].

The questionnaire’s final version was distributed to three successive classes (2008–2010) of 6th-year students at the Hebrew University – Hadassah School of Medicine, Jerusalem, Israel and the 2010 class of the Ben Gurion University School of Medicine, Be’er Sheva, Israel. These two schools were chosen because they are the investigators’ home institutions. This allowed them to insure a high response rate.

Portions of these data have been reported in a methodological validation (5). This study was approved by the Institutional Review Board of the Hadassah Medical Organization.

For statistical analysis, all the points on the Likert Scale were treated as continuous variables. The Likert Scale data were subjected to both hierarchal cluster analysis and factor analysis. In the latter, varimax rotation was used with set eigenvalues of ≥1.0. When reported as categorical data, the 5-points of the Likert Scale were reduced to three categories (the percentages of the responses from the two points representing negative responses were added together as were the percentages of two points representing positive responses plus the middle point). The percentage of responses for each of the three categories was calculated.

Analysis focused on two issues. (1) The students’ perceptions of each of the six specialties and how the perception patterns differed between the various specialties. (2) The differences between the selection criteria and perceptions of students considering and not considering pursuing a career in each of the specialties. For this analysis the replies of the students who chose one of the two negative points on the 5-point Likert Scale to the query “are you considering a career in this specialty” were compared to those of the students who chose one of the two positive points; i.e. the neutral choice was not included in the analysis.

These data were managed with Excel 2003® (Microsoft Inc., Redmont, WA) and analyzed using Systat Version 12® (Systat Inc. San Jose, CA). Categorical data are presented as frequency distributions. Chi-squared analysis was performed for binomial responses. Two-tailed Student’s t-tests compared continuous variables and Bonferroni corrections were employed for multiple comparisons. Based on prior investigations demonstrating significant differences in interests and selection criteria between the genders [9,10], it was decided during the study’s design phase to separately evaluate data from male and female students.

## Results

A response rate of 66% (234 of 355 questionnaires) was obtained. Of the respondents, 52% were women and 53% were single. The male students were older (p<0.001) than the female ones.

**Figure 1:**
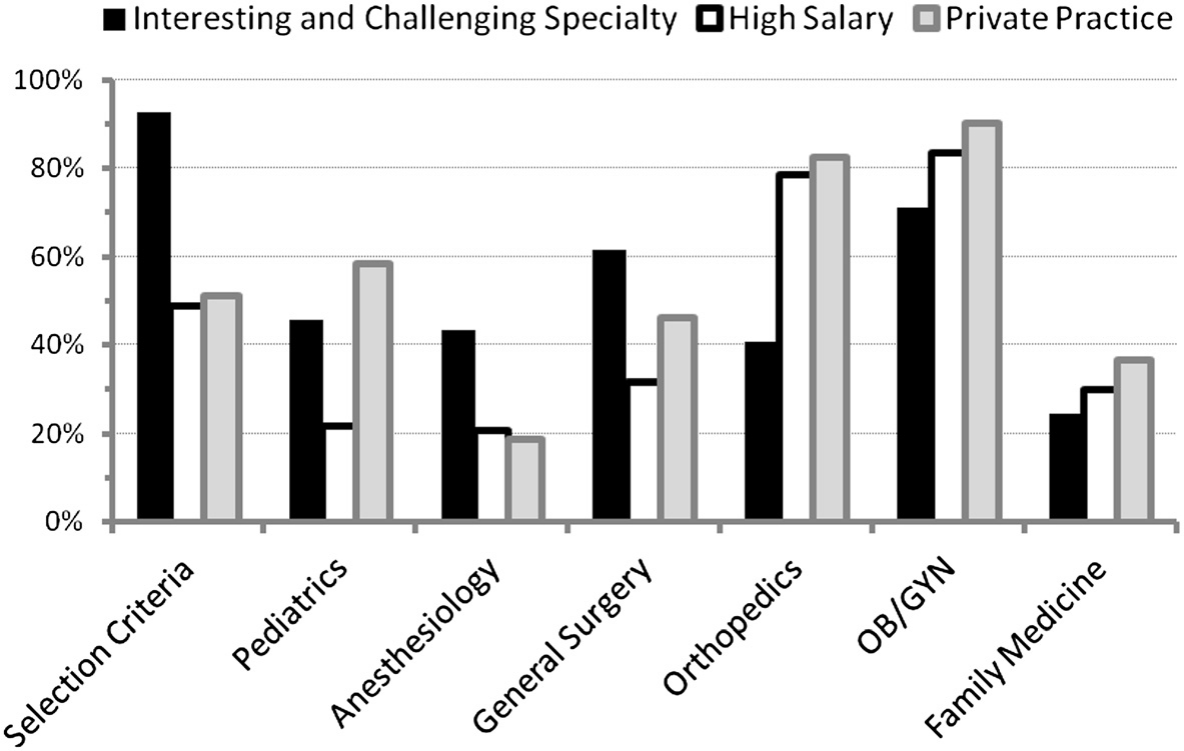
**Students’ selection criteria versus their perceptions of the six specialties.** Three selection criteria are contrasted with students’ perceptions of the six specialties. An interesting and challenging specialty was reported to be an important/very important selection criterion by almost all the students. Despite family medicine being considered by only 23% of the students as interesting/challenging it was selected as a possible career choice by 19%.

**Figure 2:**
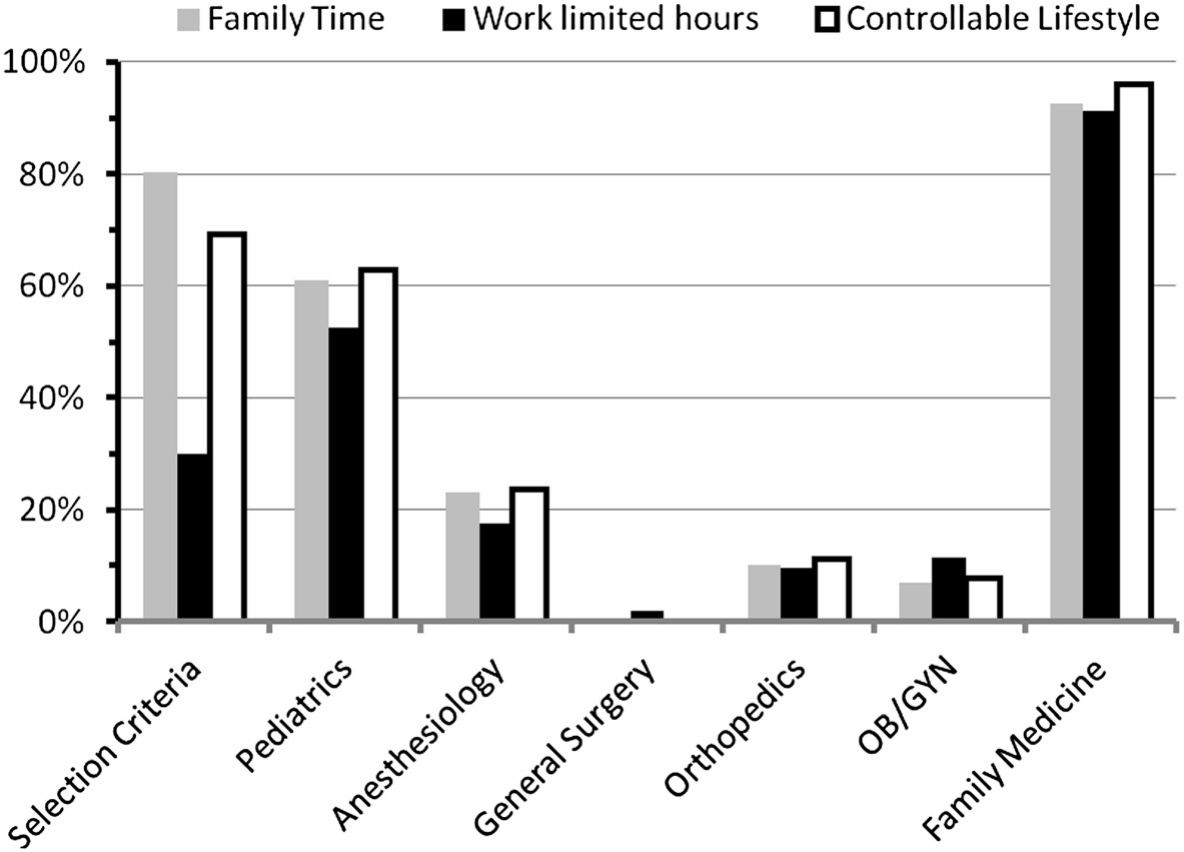
**Students’ selection criteria versus their perceptions of the six specialties.** Three of the selection criteria are contrasted with the students’ perceptions of corresponding factors. Both a controllable lifestyle and family time were considered important/very important selection criteria by many students. Pediatrics and family medicine were considered to afford both controllable lifestyles and family time.

As indicated in Figures 1 and 2, the selection criteria rated most highly were “interesting and challenging specialty”, “family time”, and “controllable lifestyle”. The criterion rated lowest was “work limited hours”, while “high salary” and “private practice” received intermediate ratings.

The medical students’ perceptions of all six specialties are found in Table 1, which displays the percentage of agree/highly agree replies on the 5-point Likert Scale. Each specialty had a distinct profile. Pediatrics and obstetrics/gynecology were the specialties most often reported as being under positive consideration for a career both by the students themselves and by their peers. Anesthesiology and general surgery were least often being positively considered as careers by the students and both were thought to be in workforce crises. There were no differences in the findings between the two schools.

**Table 1:** Medical students’ perceptions of the six specialties (n = 234)

	**Pediatrics**	**Anesthesiology**	**General**	**Orthopedic**	**OB/GYN**	**Family**
			**Surgery**	**Surgery**		**Medicine**
Interesting and challenging specialty	46%	43%	62%‡	41%†	71%§*‡†	25%*‡†ª
Advanced specialty	36%	44%*	35%*‡	52%*†	69%*†	23%*‡†§ª
Boring specialty	21%	35%*	14%‡	30%*†	7%*‡†§	48%*†ª
Stressful specialty	27%	74%*	94%*‡	48%*‡†	81%*†§	13%*‡†§ª
Affords controllable lifestyle	63%	24%*	0%*‡	11%*†	8%*†	96%*‡†§ª
Possible to work limited hours	53%	17%*	2%*‡	10%*†	12%*†	91%*‡†§ª
Allows for family time	61%	23%*	0%*‡	10%*†	7%*†	93%*‡†§ª
Long working hours	25%	62%*	97%*‡	75%*†	86%*‡†	6%*‡†§ª
Prestigious specialty (population)	33%	5%*	78%*‡	49%*‡†	71%*‡§	7%*†§ª
Prestigious specialty (colleagues)	15%	10%*	46%*‡	34%*‡†	61%*‡†	2%*‡†§ª
Opportunity for private practice	58%	19%*	46%*‡	83%*‡†	90%*‡†	37%*‡†§ª
High salary	22%	21%*	32%*‡	79%*‡†	84%*‡†	30%‡§ª
Reasonable ratio of lifestyle vs income	58%	20%*	6%*‡	53%‡†	45%‡†	78%*‡†§ª
Academic opportunities	45%	46%	58%	46%	78%*‡†	8%*‡†§ª
Specialty other students positively consider	67%	1%*	6%*‡	50%*‡†	76%‡†§	32%*‡†ª
Specialty in crisis	7%	97%*	74%*‡	3%‡†	3%‡†	16%‡†§ª
*Specialty I am positively considering*	35%	9%*	20%*	16%*	34%‡†§	19%*‡ª
* p<0.001 vs pediatrics		Values are the percentage of agree/highly agree answers on a 5 point-Likert Scale				

‡ p<0.001 vs anesthesiology.

† p<0.001 vs surgery.

§ p<0.001 vs orthopedics.

ª p<0.001 vs OB/GYN.

Hierarchal cluster analysis revealed the following cluster of criteria: controllable lifestyle, family time, and working limited hours. Factor analysis included the following factors: (1) working limited hours and family time with long working hours as a reciprocal or inverse member^b^ (2) high salary and specialty being highly considered by colleagues; and (3) long working hours with reciprocal member, the ratio of lifestyle to income.

Only a few differences were found between the perceptions of male and female students of each of the specialties (Table 2). The women students had a more positive view of pediatrics and family medicine than the men. Conversely, the men had a more positive perception of orthopedic surgery. Perceiving a specialty as interesting and challenging was a major determinant of positively considering a specialty.

**Table 2:** Gender differences in the perceptions of the six specialties (Women: n=122; Men: n=112)

	**Males**	***Females***	**Males**	***Females***	**Males**	***Females***
	**Pediatrics**	***Pediatrics***	**Anesthesiology**	***Anesthesiology***	**General**	***General***
					Surgery	*Surgery*
Interesting and challenging specialty	38%	53%*	45%	*42%*	73%	51%*
Advanced specialty	34%	*37%*	39%	*48%*	37%	*33%*
Boring specialty	31%	12%*	37%	*34%*	11%	*16%*
Stressful specialty	27%	*26%*	70%	*78%*	95%	*94%*
Affords a controllable lifestyle	62%	*64%*	28%	*20%*	0%	*0%*
Possibility to work limited hours	56%	*50%*	21%	*14%*	1%	*3%*
Allows for family time	60%	*62%*	29%	*18%*	0%	*0%*
Long working hours	30%	*21%*	57%	67%*	99%	*96%*
Prestigious specialty (population)	30%	*36%*	5%	*4%*	75%	*80%*
Prestigious specialty (colleagues)	17%	*13%*	10%	*11%*	50%	*43%*
Opportunity for private practice	59%	*58%*	18%	*22%*	51%	*42%*
High salary	23%	*21%*	19%	*22%*	30%	*33%*
Reasonable ratio of lifestyle to income	57%	*58%*	22%	*19%*	4%	*8%*
Academic opportunities	50%	*41%*	45%	*47%*	63%	*55%*
Specialty in crisis	8%	*6%*	97%	*96%*	81%	*68%*
Specialty other students positively consider	69%	*66%*	0%	1%*	7%	*5%*
Specialty I am positively considering	24%	45%*	14%	5%*	32%	9%*
	Males	*Females*	Males	*Females*	Males	*Females*
	Orthopedic	*Orthopedic*	Obstetrics/	*Obstetrics/*	Family	*Family*
	Surgery	*Surgery*	Gynecology	*Gynecology*	Medicine	*Medicine*
Interesting and challenging specialty	53%	30%*	64%	78%*	22%	27%*
Advanced specialty	58%	46%*	63%	76%*	22%	*23%*
Boring specialty	23%	*36%*	7%	*8%*	52%	44%*
Stressful specialty	51%	*45%*	78%	*83%*	13%	14%*
Affords a controllable lifestyle	14%	*9%*	8%	*8%*	96%	*95%*
Possibility to work limited hours	9%	*10%*	10%	*13%*	90%	*93%*
Allows for family time	11%	*9%*	6%	*8%*	91%	*94%*
Long working hours	79%	*72%*	83%	*88%*	6%	*5%*
Prestigious specialty (population)	48%	*50%*	64%	78%*	6%	*9%*
Prestigious specialty (colleagues)	34%	*35%*	53%	69%*	3%	*1%*
Opportunities for private practice	87%	*78%*	92%	*87%*	36%	*37%*
High salary	80%	*77%*	87%	*69%*	30%	*31%*
Reasonable ratio of lifestyle to income	55%	*51%*	48%	*42%*	78%	*77%*
Academic opportunities	48%	*45%*	79%	*76%*	9%	*10%*
Specialty in crisis	3%	*4%*	3%	*3%*	17%	*15%*
Specialty other students positively consider	58%	44%*	75%	*78%*	27%	36%*
Specialty I am positively considering	28%	6%*	32%	*36%*	13%	25%*

* vs males p <0.05.

Values are the percentage of agree/highly agree answers on a 5 point-Likert Scale.

The differences between the perceptions and selection criteria of students considering and not considering pursuing a career in each of the six specialties are found on Table 3. The research not only provides insight into the students’ views of each of the specialties, but also the interactions between their selection criteria, perceptions, and interest in pursuing a specific specialty as a career. For example, a specialty providing an opportunity for private practice was deemed an important/very important selection criterion by 51% of respondents (Figure 1), while orthopedic surgery and obstetrics/gynecology were perceived by 83% and 90%, respectively, as permitting private practice (Table 1). Furthermore, when students positively considering these two specialties as careers were compared to those not positively considering them, the former significantly more often rated the selection criterion, opportunity to practice privately, as important/very important (Table 3). Conversely, family medicine was perceived by only 37% of respondents as permitting an opportunity for private practice (Table 3). Furthermore, only 34% of those considering the specialty rated an opportunity for private practice as an important/very important selection criterion versus 56% who were not considering it.

**Table 3:** Perceptions and selection criteria of students considering and not considering pursuing a career in each of the specialties

	**Pediatrics**			**Anesthesiology**			**General surgery**		
	**Yes**	**No**		**Yes**	**No**		**Yes**	**No**	
Specialty I am positively considering	(n=75)	(n=99)		(n=21)	(n=164)		(n=41)	(n=142)	
	32%	42%		9%	70%		18%	61%	
Interesting and challenging specialty	76%	22%	p<0.001	90%	34%	p<0.001	90%	51%	p<0.001
Advanced specialty	57%	23%	p<0.001	67%	40%	p<0.003	38%	33%	NS
Boring specialty	7%	36%	p<0.001	21%	42%	p=0.003	5%	19%	p<0.001
Stressful specialty	36%	18%	p<0.023	84%	72%	NS	95%	94%	NS
Controllable lifestyle	68%	66%	NS	35%	26%	NS	0%	0%	NS
Work limited hours	51%	58%	NS	26%	19%	NS	2%	2%	NS
Allows family time	64%	63%	NS	37%	24%	NS	0%	0%	NS
Long working hours	57%	23%	p<0.001	67%	40%	p<0.003	38%	33%	NS
Prestigious specialty (population)	36%	27%	p<0.05	11%	4%	NS	76%	79%	NS
Prestigious specialty (colleagues)	22%	10%	p=0.03	26%	8%	NS	63%	42%	p=0.048
Opportunities for private practice	59%	60%	NS	32%	16%	NS	54%	42%	NS
High salary	20%	21%	NS	21%	21%	NS	34%	31%	NS
Ratio of lifestyle vs income	65%	54%	NS	28%	20%	NS	5%	6%	NS
Academic opportunities	45%	45%	NS	42%	43%	NS	68%	55%	NS
Specialty in crisis	8%	9%	NS	95%	97%	NS	73%	74%	NS
Requested specialty	77%	56%	p=0.007	0%	0%	NS	17%	4%	NS
**Selection criteria**									
Bedside specialty	80%	66%	p=0.004	63%	71%	NS	63%	76%	NS
Direct aid to patients	77%	77%	NS	76%	78%	NS	85%	74%	NS
Direct patient care	69%	55%	p=0.02	43%	65%	NS	44%	66%	p=0.009
Time for childrearing	87%	58%	p<0.001	71%	72%	NS	49%	81%	p<0.001
Family time	92%	69%	p<0.001	80%	80%	NS	56%	88%	p<0.001
Only daytime work	44%	18%	p<0.001	19%	29%	NS	7%	38%	p<0.001
Work outside the hospital	11%	8%	NS	14%	11%	NS	2%	12%	p<0.001
Long-term care	44%	32%	NS	24%	46%	NS	15%	49%	p=0.002
Family orientation	45%	28%	p=0.002	26%	39%	NS	15%	42%	p=0.002
Immediate satisfaction	49%	61%	NS	79%	50%	NS	90%	42%	p<0.001
Operating room time	23%	53%	p<0.001	29%	38%	NS	71%	24%	p<0.001
Performing procedures	35%	61%	p<0.001	57%	46%	NS	90%	33%	p<0.001
Controllable lifestyle	68%	65%	NS	67%	69%	NS	46%	79%	p=0.004
“Action” specialty	25%	48%	p<0.002	43%	36%	NS	73%	25%	p<0.001
Without long-term care	11%	13%	NS	24%	10%	NS	12%	11%	NS
On-cal as an attending	41%	33%	NS	33%	35%	NS	44%	34%	p=0.008
High salary	47%	48%	NS	62%	45%	NS	49%	46%	NS
Private practice	47%	56%	NS	38%	50%	NS	51%	49%	NS
Prestigious specialty	8%	15%	p<0.003	5%	12%	p<0.037	27%	8%	p=0.002
Prestigious specialty	8%	14%	NS	5%	10%	NS	24%	7%	p=0.007
Interesting specialty	91%	93%	NS	81%	93%	NS	93%	91%	NS
Gender (Female)	68%	42%	p<0.001	29%	52%	p<0.043	24%	62%	
Marital status (Single)	47%	55%	NS			NS	52%	59%	NS
	**Orthopedic surgery**			**Obstetrics/gynecology**			**Family medicine**		
	**Yes**	**No**		**Yes**	**No**		**Yes**	**No**	
Specialty I am positively considering	(n=34)	(n=155))		(n=74)	(n=102)		(n=41)	(n=137)	
	15%	66%		32%	44%		18%	59%	
Interesting and challenging specialty	88%	27%	p<0.001	93%	55%	p<0.001	68%	9%	p<0.001
Advanced specialty	68%	47%	p=0.036	84%	60%	p<0.001	29%	23%	NS
Boring specialty	6%	39%	p<0.001	3%	12%	p<0.001	18%	65%	P<0.001
Stressful specialty	44%	50%	NS	88%	80%	NS	12%	15%	NS
Controllable lifestyle	26%	7%	p=0.011	9%	7%	NS	100%	94%	NS
Work limited hours	12%	7%	NS	11%	9%	NS	93%	90%	NS
Allows family time	18%	8%	p=0.043	7%	6%	NS	93%	92%	NS
Long working hours	85%	74%	NS	89%	84%	NS	15%	4%	P=0.028
Prestigious specialty (population)	59%	47%	NS	71%	67%	NS	8%	7%	NS
Prestigious specialty (colleagues)	26%	38%	NS	64%	57%	NS	0%	3%	NS
Opportunities for private practice	97%	79%	p<0.001	88%	88%	NS	30%	41%	NS
-High salary	82%	77%	NS	84%	81%	NS	33%	31%	NS
Reasnable ratio of lifestyle vs. income	62%	50%	p=0.042	51%	39%	NS	93%	73%	p=0.004
Academic opportunities	62%	42%	NS	81%	74%	NS	15%	6%	P=0.012
Specialty in crisis	6%	3%	NS	1%	4%	NS	26%	15%	NS
Requested specialty	62%	45%	p=0.018	73%	73%	NS	40%	28%	NS
**Selecion criteria**									
Bedside specialty	53%	77%	NS	74%	74%	NS	85%	72%	p=0.036
Direct aid to patients	76%	77%	NS	76%	75%	NS	80%	79%	NS
Direct patient care	35%	68%	p=0.004	57%	65%	NS	78%	57%	p=0.042
Time for childrearing	50%	77%	p=0.023	66%	73%	NS	95%	63%	p<0.001
Family time	62%	84%	p=0.016	76%	79%	NS	95%	74%	p<0.001
Only daytime work	15%	34%	p=0.001	26%	29%	NS	51%	20%	p<0.001
Work outside the hospital	6%	12%	p=0.046	7%	12%	NS	24%	5%	p<0.001
Long-term care	15%	45%	p<0.001	41%	35%	NS	68%	28%	p<0.001
Family orientation	15%	39%	p=0.003	36%	31%	NS	70%	24%	p<0.001
Immediate satisfaction	79%	46%	p<0.001	70%	48%	p<0.001	34%	63%	p=0.003
Operating room time	74%	28%	p<0.001	53%	33%	p<0.001	17%	46%	p<0.001
Performing procedures	85%	39%	p<0.001	58%	41%	NS	24%	55%	p<0.001
Controllable lifestyle	68%	70%	NS	59%	74%	NS	83%	62%	NS
“Action” specialty	59%	31%	p<0.001	45%	34%	p<0.008	15%	48%	p<0.001
Without long-term care	9%	12%	NS	11%	11%	NS	10%	12%	NS
On-cal as an attending	38%	37%	p=0.029	37%	38%	NS	27%	44%	p=0.006
High salary	62%	43%	p=0.024	53%	42%	NS	46%	47%	NS
Private practice	62%	45%	p<0.001	58%	43%	p<0.004	34%	56%	p=0.022
Prestigious specialty (colleagues)	18%	9%	NS	13%	12%	NS	3%	18%	p=0.002
Prestigious specialty (population)	53%	48%	NS	11%	11%	NS	2%	16%	p=0.039
Interesting specialty	91%	92%	NS	92%	91%	NS	88%	93%	NS
Gender (female)	18%	63%	p<0.001	55%	47%	NS	68%	46%	p=0.011
Marital status (single)	68%	50%	p=0.021	43%	59%	NS	44%	55%	NS

Another instance of such interaction was with the selection criterion “work only daytime hours” (Figure 2), reported as important/very important by 30% of the students. Ninety percent and 53% of students perceived that family medicine and pediatric specialists, respectively, had the possibility to “work limited hours”. When students positively considering these two specialties as careers were compared to those who were not positively considering them, the former significantly more often rated the selection criteria, “work only daytime hours”, as important/very important (Table 3). Alternately, few students positively considering careers in general and orthopedic surgery perceived the specialties as being conducive to working limited hours (2% and 10% of students respectively), and were significantly less likely to consider the selection criteria “work only daytime hours” as important/very important than the rest of the students.

## Discussion

This study provides Israeli healthcare leaders with information about how a group of Israeli medical students perceive six of the nine specialties with the greatest number of specialists [11]. The concrete opinions the students had about each of the six specialties elucidates medical specialty perception patterns prevalent among Israeli students, e.g. anesthesiology was universally unpopular among both genders and was overwhelmingly perceived as suffering from a workforce crisis. Moreover, this study provides further insights into these perceptions by simultaneously examining how the students’ selection criteria matched their perceptions. When assessed from the marketing research perspective, these data provide the vendors (i.e. healthcare system administrators, department chairs and residency program directors) with objective information on whether certain aspects of a specialty’s working conditions, remuneration, or clinical spectrum need to be changed to better align them with the students’ selection criteria.

For instance, practically no students perceived general surgery as providing a controllable lifestyle or family time, which were reported as important/very important selection criteria by 69% and 80% of respondents, respectively. Additionally, <7% opined that the specialty provides a reasonable relationship between lifestyle and income. Therefore, it is not surprising that 74% perceived general surgery as being plagued by a workforce crisis and reported that only 6% of their colleagues were considering it as a career.

These results reflect the notion that it is extremely difficult to attract potential buyers (students) to a product (specialty) they consider unappealing. In a market environment, unappealing products are usually modified to meet consumers’ expectations, subjected to a different marketing/sales strategy, or withdrawn from the market [12]. The latter option is not relevant in the medical specialty arena. Therefore, when marketing the various specialties to medical students, the features perceived by them as causing some specialties to be unattractive might need modification to improve their appeal. Obviously, such changes may not be easily implemented but could be essential for specialties unable to attract sufficient students to their residency programs. For example, in the United States and other countries, efforts to modify the general surgery specialty have met with some successes in recruiting more residents, especially female ones [13,14].

In many countries, primary care specialties, such as family medicine and pediatrics, are unpopular among medical students due to uncontrollable lifestyles and the poor relationship between lifestyle and income [15-17]. This situation is attributed to relatively low remuneration and solo practices, often in rural areas, leading practitioners to be at the beck and call of their patients [18]. In some of these countries anesthesiology is a popular specialty because it provides a controllable lifestyle with defined working hours and excellent remuneration [14,19]. The present study shows that in Israel the opposite is true. Specifically, family medicine was perceived as affording a controllable lifestyle, family time, and reasonable ratio of lifestyle to income along with the possibility of working limited hours. Pediatrics was similarly perceived. Both specialties were not considered to be experiencing workforce problems and were more attractive to female students than to male students, with 25% and 45% of female students positively considering careers in family medicine and pediatrics, respectively. This popularity is attributed to the characteristics of the Israeli medical system where such practitioners are largely employees of health maintenance organizations working fixed hours with minimal on-call duties and no in-patient responsibilities.

Anesthesiology was deemed by 97% of the respondents to be suffering a workforce crisis and by only 0.5% of respondents as being positively considered as a career by of their colleagues. Although anesthesiology was perceived as interesting and challenging by 43% of students, on par with pediatrics and orthopedic surgery, it was perceived by the majority of students as not providing a controllable lifestyle, high salary, the possibility of working limited hours, or allowing for family time. Furthermore, it was perceived as requiring long working hours and as having an unfavorable ratio of lifestyle to income. These results are not unexpected, given that Israeli anesthesiology is plagued by a perennial shortage of physicians leading to a vicious cycle of long working hours and many on-call duties, thereby further discouraging medical students from joining anesthesiology residency programs [20,21]. However, this study points to some features of the specialty that could be remediated in order to break this cycle. These include improved working conditions, compensation, and image. Corrective measures in other countries, aimed at similar issues, have successfully attracted medical students to the specialty [19].

There were distinct differences between how the student’s perceived each of the six specialties (Table 1); however, within each of the specialties there were few disparities between the responses of male and female students (Table 2). As an example, 63% of the respondents reported that pediatrics affords a controllable lifestyle, while only 8% reported that obstetrics/gynecology affords the same. Yet, in both instances there were no differences between the opinions of male and female students. In fact, most of the gender differences were in response to the questions of whether the specialty was boring or interesting and challenging. For example, a higher proportion of women perceived pediatrics and family medicine to be interesting and challenging, while a higher proportion of men thought that general and orthopedic surgery were interesting and challenging. Correspondingly, a higher proportion of women considered pediatrics and family medicine as specialties they were positively considering while a higher proportion of men were positively considering orthopedic and general surgery.

The importance of a specialty being interesting and challenging recurs throughout this study, with over 92% of the student’s reporting it as being an important/very important selection criteria. Further, being interesting and challenging is the most consistently statistically significant perception when the opinions of those considering were compared to those not considering pursuing each of the 6 specialties (Table 3). However central “interest” is to selecting a career, it is a nebulous concept that is difficult to concretely define. Interest is a conglomeration of many factors including personality; intellectual ability and aptitude; manual skill; physical and emotional dispositions; and willingness to work in particular environments [3,22]. Interest also often correlates with gender, with men generally preferring to work with things and women with people [23,24]. Therefore, subsequent studies are needed to more deeply probe the determinants of interest as they relate to medical specialty selection and students’ perceptions of the various specialties. Such studies promise to provide further insight into how specialty selections are made.

### Strengths and limitations

This is among the few studies to examine Israeli medical students’ perceptions of some of the healthcare system’s key medical specialties. It provides both insight into the students’ views of each of the specialties and how their selection criteria, perceptions, and interest in pursuing a specialty as a career interact. A limitation of this study is that since it measures perceptions, it is possible that students do not base answers on objective information but, rather, on impressions, biases, and incomplete information. However, it is very important to study perceptions, as they are the basis of many decisions in life. Moreover, we did not elicit the source of these perceptions to determine the basis on which they were formed, a topic that should be studied in the future. Finally, the questionnaires were anonymous so it was not possible to assess the characteristics of the non-responders. However, we do know that the male:female ratio of the responders reflects the male:female ratio of the medical school classes.

## Conclusions

The Israeli healthcare system is continuously faced with the predicament of balancing the ever-increasing demand for a wide spectrum of clinical services with a sufficient supply of appropriately trained professionals to provide these services. However, the physician workforce is facing both an impending shortage of physicians and a maldistribution of physicians among the various specialties. The shortage has been officially recognized, leading to larger class sizes in existing medical schools and the opening of an additional medical school. However, only recently have some initiatives been taken to remedy the specialty distribution problem. Incentives were provided in the 2011 collective bargaining agreement which increased remuneration for specialties suffering workforce shortfalls, including general surgery and anesthesiology, and provided one-time monetary grants to those entering residencies in these specialties as well ongoing salary differentials. However, these initiatives were not based on objective data, but on the negotiators and their advisors opinions of what they thought would attract more medical students and interns to specialties with workforce shortages. This subjective approach is fraught with risks since it does not necessarily promise a solution to the problem. From the marketing research viewpoint, it is akin to developing a product without asking potential customers whether they would consider buying it.

The present study demonstrates the use of an objective approach to examining workforce problems, namely, how the selection criteria of the “consumers” match their perceptions of specific specialties. These results are the initial phase of a marketing research study and provide data for designing the next phase which will examine in greater detail the “customer’s” positive and negative opinions of the “product”. This second phase leads to further phases that elicit “customer’s” opinions on proposed improved versions of the “product” that were designed using the previously obtained perceptions and opinions. This type of objective approach is generally unappealing to medical workforce administrators because it requires substantial personnel and expertise to perform the analyses. However, this type of research is important so that healthcare leaders can understand the younger generation’s behavioral patterns and aspirations that focus on working conditions that contribute to controllable lifestyles and quality family time [25].

Besides demonstrating that gathering objective data on the opinions and perceptions of Israeli medical students provides a way to better understand physician workforce dynamics, this study also provides the Israeli healthcare leadership with a detailed assessment of Israeli medical students’ perceptions of some of the major specialties. This information can be employed to tailor incentives to specifically attract students to specialties with workforce shortages. Workforce shortages in anesthesiology and general surgery are not surprising, given the poor opinions of those specialties reported from the students. These data provide evidence for the need for more than just one-time financial grants and small increases in salary to attract students to anesthesiology. It shows the need to improve the relationship of lifestyle to remuneration. This study also shows that the general surgery leadership needs to address the perceptions and realities that exist including, hard work, long hours, and an uncontrollable lifestyle. Finally, the information generated could also be used to design active interventions, such as marketing campaigns, to better recruit students to problematic specialties [18,26]. Studies similar to the current one should thus be performed on a regular basis using similar methodology to study other specialties in addition to the six included in the present study to assist in understanding residency selection trends among the medical students in all 5 of the Israeli medical schools.

## Endnotes

^a^Internal Medicine was not chosen because it is usually a “pass through” residency i.e. a way-station on the way to sub-specialization. Only a minority of Israeli internal medicine residents do not sub-specialize. Therefore, the responses we would receive might not properly examine general internal medicine as a specialty but might also reflect the student’s view of a possible subspecialty. We are in the process of developing a questionnaire to specifically study interest in general internal medicine vs interest in subspecialty training, but it is difficult to obtain a clear separation.

^b^The mathematical definition of reciprocal is inverse, opposite. In the present study reciprocal is used in factor analysis to designate a factor that relates in the opposite. For example, the manuscript relates that one factor was: “Working limited hours and family time. Long working hours was a reciprocal member”. Therefore, working limited hours and long working hours were identified as being inversely related to each other. To clarify the issue we added the word “inverse” in parenthesis after reciprocal.

## Competing interests

The authors declare that they have no competing interests.

## Authors’ contributions

CW concept, study design, data collection and analysis and manuscript writing. HT concept, questionnaire design, data collection, review and revision of manuscript. RYZR – study design, data collection and analysis, revision of manuscript. YGW questionnaire design, study design, data analysis and interpretation, review and revision of manuscript. UE study design, review and revision of manuscript. AA design, review and revision of manuscript. JES concept, study design, review and revision of manuscript. All authors read and approved the final manuscript.
